# Supplementary material for: BlurryScope enables compact, cost-effective scanning microscopy for HER2 scoring using deep learning on blurry images
Source: NPJ Digit Med. 2025 Aug 6;8:506. doi: 10.1038/s41746-025-01882-x (PMC12325710; doi:10.1038/s41746-025-01882-x)
Supplement: Supplementary file 1 — Supplementary Information [file 41746_2025_1882_MOESM1_ESM.pdf]

Supplementary Materials

BlurryScope enables compact, cost-effective scanning microscopy for HER2 scoring using deep learning on blurry images

Michael John Fanous[1], Christopher Michael Seybold[2,#], Hanlong Chen[1,3,4,#], Nir Pillar[1,3] and Aydogan Ozcan[1,3,4,5,*]

[1]Electrical and Computer Engineering Department, University of California, Los Angeles 90095 CA, USA
[2]Mathematics Department, University of California, Los Angeles 90095 CA, USA
[3]Bioengineering Department, University of California, Los Angeles 90095 CA, USA
[4]California NanoSystems Institute (CNSI), University of California, Los Angeles 90095 CA, USA
[5]Department of Surgery, David Geffen School of Medicine, University of California, Los Angeles 90095 CA, USA

[#]C.M.S and H.C: these authors contributed equally to this work.

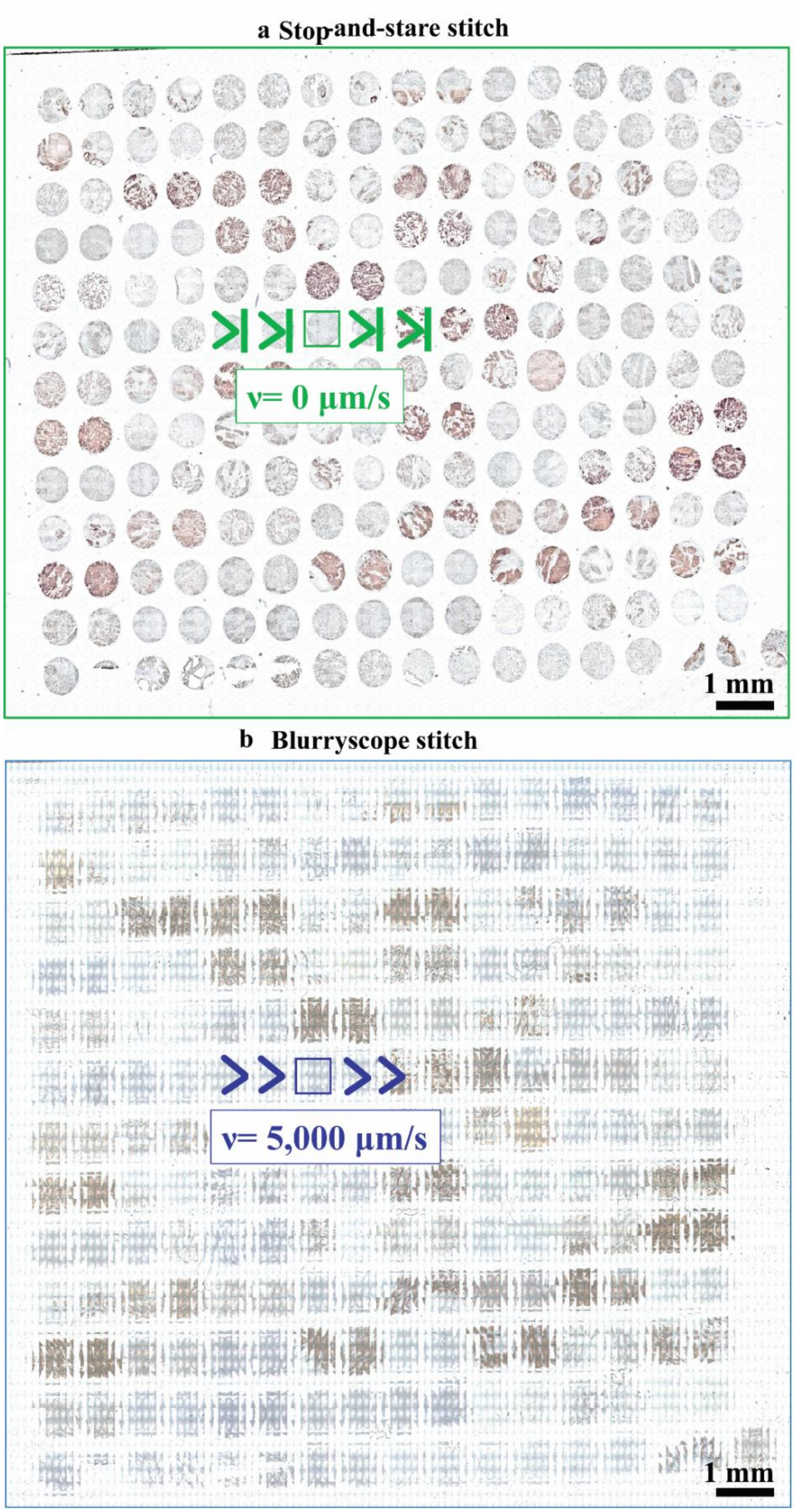
a Stop-and-stare stitch
v= 0 µm/s
1 mm
b Blurryscope stitch
v= 5,000 µm/s
1 mm

Supplementary Figure 1. Comparison between stitched TMA slide results using (a) the classic "stop and stare" approach, and (b) the zigzag scanning configuration of BlurryScope.

A comparison between two different stitching results from the scan of a whole-slide tissue microarray (TMA). The top portion of Supplementary Figure 1 shows a stitched TMA slide where individual image tiles were captured while the stage remained stationary at each position, ensuring high structural integrity and minimal motion artifacts. The grid layout of circular tissue cores is well-preserved, with a clear delineation of individual samples. The bottom portion of the figure depicts a stitched TMA slide captured with a continuous scanning approach, where the stage moved at a velocity of 5,000 μm/s during image acquisition. This high-speed scanning method results in a characteristic smearing pattern, where motion artifacts are visible across the entire stitched image. Notwithstanding this smudging defect, the frames pertaining to separate cores were successfully grouped in distinct, fully separable areas.

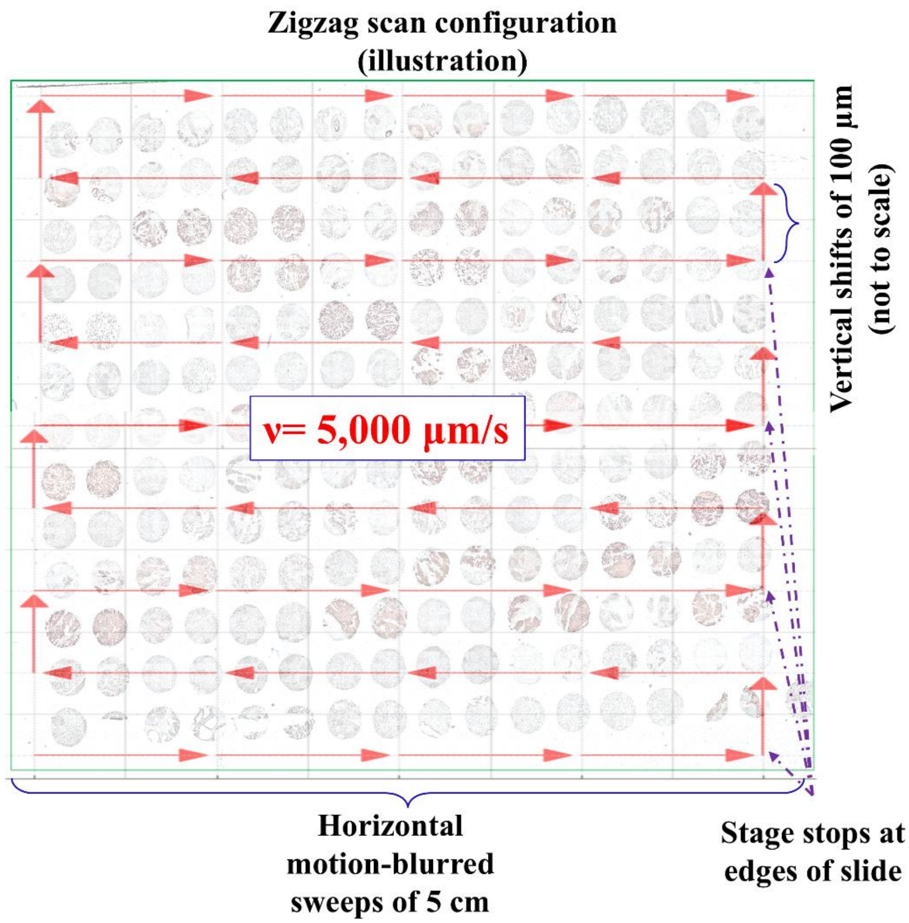

Supplementary Figure 2. Zigzag scanning configuration of BlurryScope.

The scanning strategy used by BlurryScope for rapid tissue imaging follows a zigzag motion path (red arrows in Supplementary Figure 2), continuously scanning tissue microarrays (TMAs) at a lateral speed of 5,000 μm/s. Each horizontal sweep spans 5 cm, covering a full row of tissue cores in a single pass. At the end of each horizontal sweep, the stage executes a vertical shift of 100 μm before initiating the next horizontal scan in the opposite direction. This bidirectional scanning minimizes turnaround delays, ensuring high-throughput image acquisition. The stage movement stops at the edges of the slide, maintaining a structured scanning area.

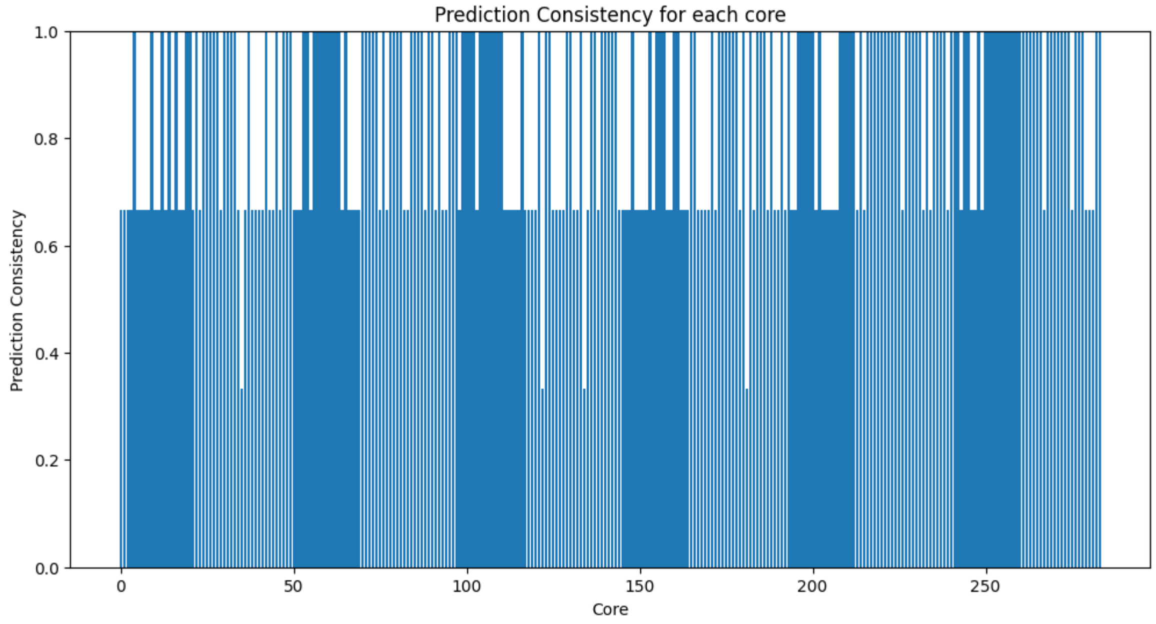

Supplementary Figure 3. Prediction consistency for HER2 classification across scanned cores using BlurryScope.

The prediction consistency across all scanned cores using BlurryScope for HER2 classification was assessed. The bar graph in Supplementary Figure 3 shows consistency levels for each core, defined as the proportion of the predictions that match the most frequent prediction (mode) across three scans per core. The results indicate high consistency across the majority of the cores (0.67 corresponds to two out of three matched scores), with some variability due to potential operational factors, such as slide placement or scan misalignments.

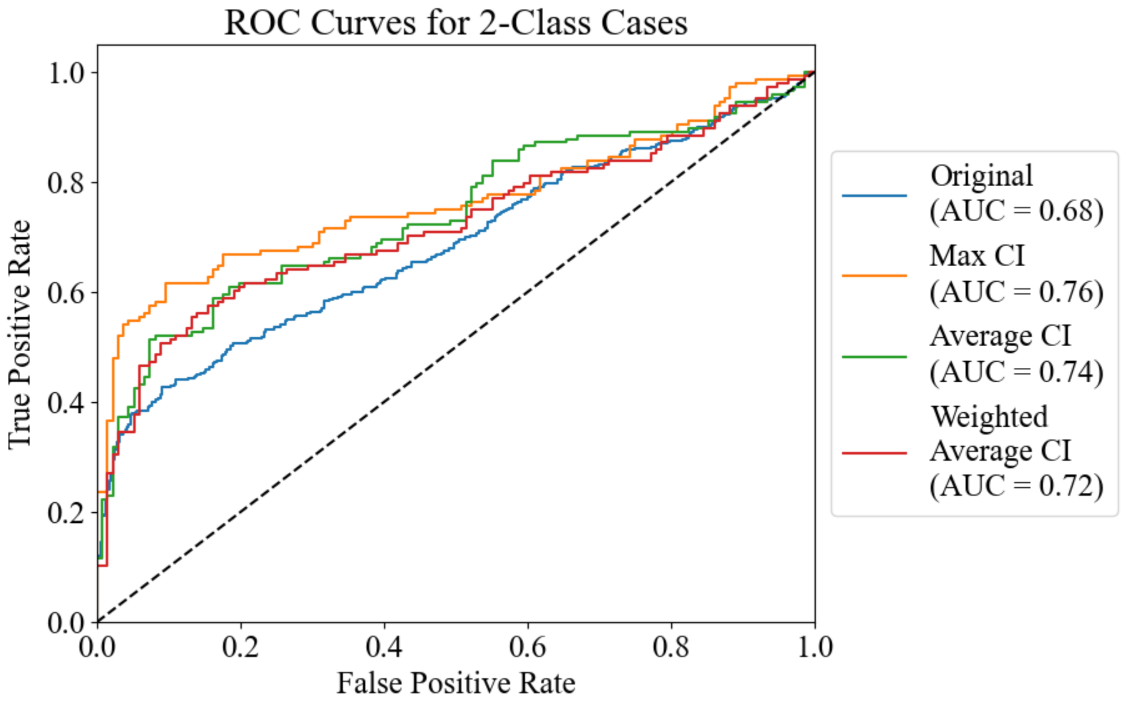

.

Supplementary Figure 4. ROC curves for the 2-class HER2 classification network using BlurryScope for different data distributions.

ROC curves for the BlurryScope 2-class HER2 classification network were evaluated. Four methods of confidence interval (CI) integration—total scans, highest CI, average (absolute, no CI weights) and weighted average CI—are compared along with their area under the curve (AUC) values. These curves demonstrate the trade-offs between sensitivity and specificity for each classification method. The maximum CI method achieves the highest AUC, indicating a superior balance between correctly identifying true positives and minimizing false positives. The weighted average and average CI methods perform comparably but lag slightly behind in overall performance.

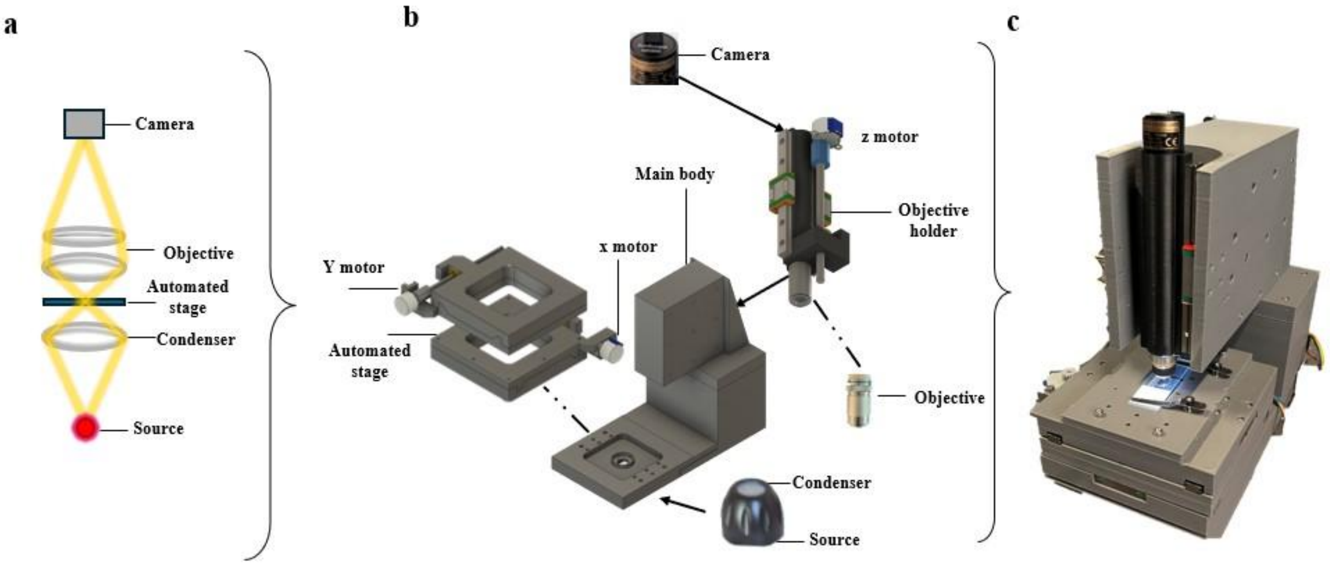

Supplementary Figure 5. BlurryScope components: (a) the optics; (b) the 3D printed components and pictures of the optical parts; (c) the final assembled system.

The optical parts of BlurryScope are shown in Supplementary Figure 5, along with pictures of 3D printed parts and connections as to how the whole system is assembled. The optical system was adapted from a dismantled M150 Compound Monocular AmScope brightfield microscope, incorporating an RGB CMOS camera into a custom 3D-printed framework. Illumination is provided by an LED source paired with a condenser unit.

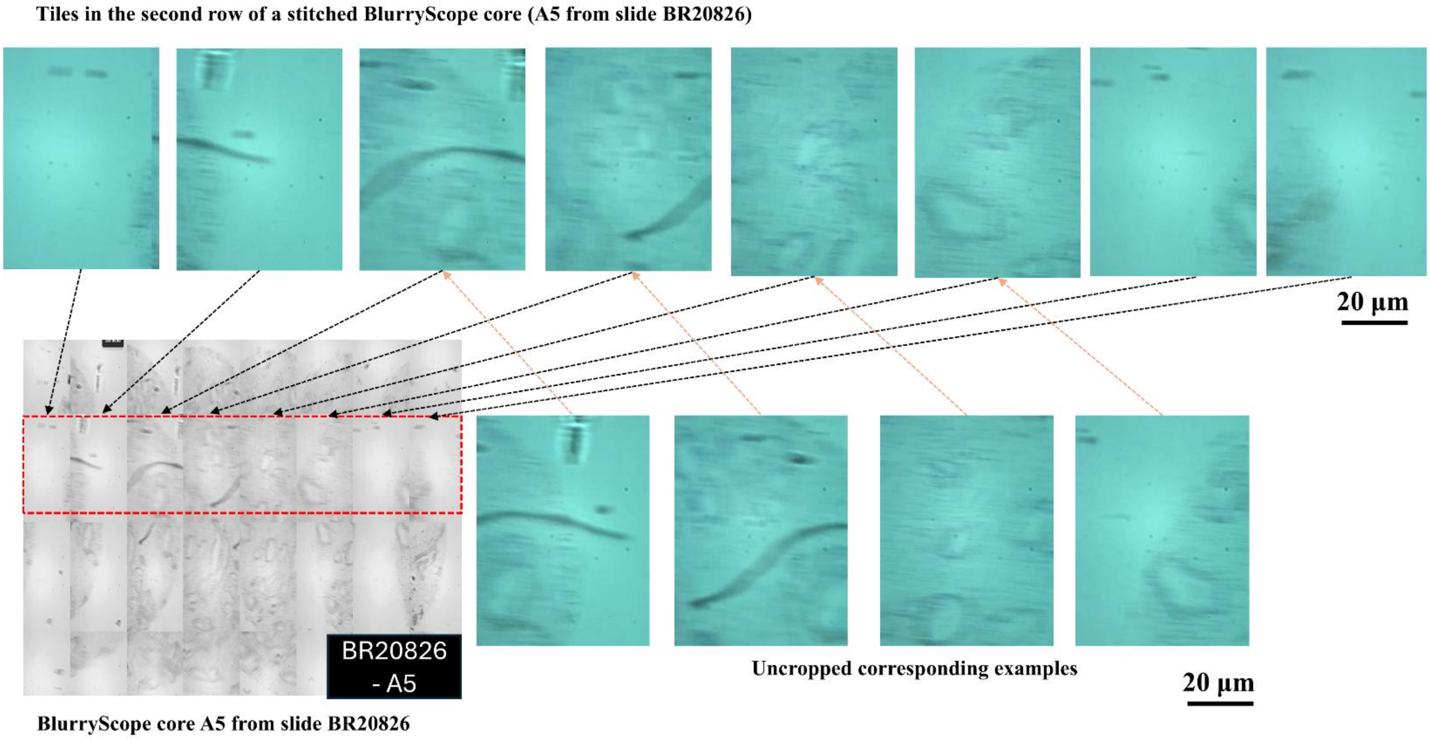

Supplementary Figure 6. BlurryScope stitch with individual tiles of a row before and after processing.

The automated tile-based stitching process for a single core captured using BlurryScope is shown in Supplementary Figure 6. The white-balanced image on the bottom left represents the stitched result, with the second row of tiles highlighted in red. The top row of images in Supplementary Figure 6 displays individual cropped tiles from this row, which were acquired separately using BlurryScope's video-style continuous acquisition method and later combined to reconstruct the full tissue core. The bottom row presents uncropped corresponding examples, showing localized details of the core frames at a size where finer structures can be observed.
